# COVID-19 lockdown impact on familial relationships and mental health in a large representative sample of Italian adults

**DOI:** 10.1007/s00127-022-02273-3

**Published:** 2022-03-28

**Authors:** Margherita Zeduri, Giacomo Pietro Vigezzi, Greta Carioli, Alessandra Lugo, Chiara Stival, Andrea Amerio, Giuseppe Gorini, Roberta Pacifici, Pierluigi Politi, Silvano Gallus, Anna Odone

**Affiliations:** 1 Department of Public Health, Experimental and Forensic Medicine, University of Pavia, via Forlanini, 2, Pavia, Italy; 2 Present Address: School of Medicine, University Vita-Salute San Raffaele, Milan, Italy; 3 Department of Clinical Sciences and Community Health, University of Milan, Milan, Italy; 4 Department of Environmental Health Sciences, Istituto di Ricerche Farmacologiche Mario Negri IRCCS, Milan, Italy; 5 Department of Neuroscience, Rehabilitation, Ophthalmology, Genetics, Maternal and Child Health (DINOGMI), Section of Psychiatry, University of Genoa, Genoa, Italy; 6 IRCCS Ospedale Policlinico San Martino, Genoa, Italy; 7 Oncologic Network, Prevention and Research Institute (ISPRO), Florence, Italy; 8 National Centre on Addiction and Doping, Italian National Institute of Health, Rome, Italy; 9 Department of Brain and Behavioral Sciences, University of Pavia, Pavia, Italy

**Keywords:** COVID-19 home confinement, Mental health, Familial relations, Italy, Cross-sectional study, Social security system

## Abstract

**Purpose:**

Benefits of national-level stay-at-home order imposed in Italy to prevent SARS-CoV-2 transmission need to be carefully weighed against its impact on citizens’ health. In a country with a strong familial culture and where welfare relies on households, confinement drastically decreased support provided by elder relatives, which may have resulted in mental health worsening.

**Methods:**

A web-based cross-sectional study (LOST in Italy) was conducted on a representative sample of Italian adults during lockdown (27th of April–3rd of May 2020). We asked 3156 subjects to report on reduced help in housework and childcare from retired parents to assess the impact of confinement on mental health, through validated scales before and during lockdown.

**Results:**

Overall, 1484 (47.0%) subjects reported reduced housework help from parents, and 769 (64.0%, of the 1202 subjects with children) diminished babysitting support. Subjects reporting reduced housework help had worsened sleep quality (multivariate odds ratio, OR = 1.74, 95% confidence interval, CI 1.49–2.03) and quantity (OR = 1.50, 95% CI 1.28–1.76), depressive (OR = 1.32, 95% CI 1.14–1.53) and anxiety symptoms (OR = 1.53, 95% CI 1.32–1.78), compared to those reporting unreduced help. Worsening in sleep quality (OR = 2.32, 95% CI 1.76–3.05), and quantity (OR = 1.80, 95% CI 1.36–2.37), depressive (OR = 1.79, 95% CI 1.39–2.31) and anxiety symptoms (OR = 1.90, 95% CI 1.48–2.46) was also associated with reduced babysitting help. Mental health outcomes were worse in subjects with poorer housing and teleworking during lockdown.

**Conclusion:**

Confinement came along with reduced familial support from parents, negatively impacting household members’ mental health. Our findings might inform evidence-based family and welfare policies to promote population health within and beyond pandemic times.

## Introduction

Coronavirus disease 2019 (COVID-19) outbreak impacted communities worldwide and, two years after the first cases, it is globally responsible for more than 373.2 million cases and more than 5.7 million deaths [1]. Italy became the first COVID-19 epicentre in Europe and, on the 9th of March 2020, it was also the first country to impose a nationwide stay-at-home order as an attempt to reduce the exponential virus spread and alleviate acute pressure on the healthcare system [2, 3]. The Italian-wide confinement order (i.e., national lockdown) lasted for 2 months until the 3rd of May and confined over 60 million people inside their homes. Italians were forced to remain at their residence home unless for basic necessities (e.g., food, shopping) or health issues. Public services (schools, shops, gyms, bars and restaurants) were closed; gatherings were forbidden either in public or private places, and a series of infection protection and control measures were recommended (mask-wearing, hand washing or hand rubbing, respiratory etiquette, physical distancing). These non-pharmacological measures are considered among the most radical ones implemented so far [4] and among the most exceptional public health measures ever imposed [5]. In compliance with these restrictions, sudden and radical changes occurred in millions of Italians’ daily life and behaviours [6]. Physical distancing and self-isolation strongly impacted social, working, and family habits, drastically reducing any form of socialisation [7]. This occurred in a country with a solid social and familial culture, where elder populations are actively involved in younger generations’ family life [8]. Within Europe, Italy holds among the highest financial transfer and social support rates from older to younger generations, with subjects > 70 years still being net givers [9]. Italy, together with other southern European countries, has “familistic” societies [10] with weaker welfare policies and governmental family benefits, as compared to Western European standards [11]. As a consequence, Italian welfare is mainly based on family support, with grandparents acting as caregivers for grandchildren on a regular basis, helping with cooking and housework, and often providing emotional and informational support [12]. As a matter of fact, in Italy, older people constitute a fundamental and beneficial resource for the provision of informal family support [10]. During COVID-19 national lockdown, since in Italy, people aged 65 years or over live alone (29.7%) or with a partner (42.2%) [13], Italian families limited their contacts with older relatives because of the stay-at-home order. Moreover, at the highest risk for COVID-19 death, older people also isolated themselves due to fear of infection [14]. Therefore, the availability of informal family care provided by elder relatives might have been substantially reduced, this adding to the burden on families already caused by schools and childcare facilities’ closures. Italian households had to deal with new needs and faced daily routines without support from retired parents. Changes in social connections in a context of stress and uncertainty might have negatively impacted households’ mental health. Without denying the benefits of national lockdown on COVID-19 control, investigating the impact of the pandemic on mental health from a multidisciplinary and familial perspective is a research priority [15, 16], even more so in a country with a demographic and social structure like the one described for Italy.

Within the ‘LOckdown and lifeSTyles in Italy’ (LOST in Italy) study [17], we explored the impact of national lockdown on selected mental health outcomes in a representative sample of Italian households, analysing the association with changes in housework and childcare help from retired parents.

## Methods

### Setting, study design and study population

This study is based on the LOST in Italy study, a web-based cross-sectional study conducted on a representative sample of 6003 Italian adults aged between 18 and 74 years [17–20]. Lombardy region, the most affected by COVID-19 in Italy, has been oversampled. From the full sample, the current study selected subjects with at least a retired parent (*n* = 3156) and, within this sub-sample, those with at least one child aged 0–14 years (*n* = 1202).

### Data sources

Doxa, the Italian branch of the Worldwide Independent Network/Gallup International Association, conducted the survey in collaboration with the Italian National Institute of Health (Rome, Italy), Mario Negri Institute for Pharmacological Research (Milan, Italy), the University of Pavia and other institutions. Survey participants were selected among the Doxa online panel, which today includes about 40,000 active panellists (subjects who have participated in at least one research over the last 12 months, with an average refresh of 25%). Data were collected during the nationwide lockdown, from the 27th of April to the 3rd of May 2020. The study protocol obtained the ethics committee’s approval (EC) of the coordinating group (i.e., EC of Besta Neurological Institute, Milan, Italy; file number 71–73, April 2020), and consent to participate was collected for all participants.

### Questionnaire and outcomes of interest

Recruited subjects were interviewed using an online self-administered questionnaire about their lifestyle habits (e.g., sleep quality and quantity), mental distress (e.g., anxiety and depressive symptoms) and quality of life before and during the lockdown. The questionnaire included information on demographic and socioeconomic variables and anthropometric data. Subjects were asked how the help for housework (e.g., bills, cleaning, housekeeping) and babysitting from retired parents had changed during lockdown (unchanged, reduced, or increased, exposure of interest).

To quantify the impact of COVID-19 lockdown on participants’ mental health, we focused on sleep quality and quantity, depressive and anxiety symptoms, quality of life, asking interviewees to answer questions with reference to both before and during the lockdown. These aspects were analysed using validated scales [20].

Sleep quality and quantity were assessed using the Pittsburgh Sleep Quality Index (PSQI) questionnaire [21]. Concerning the subjective evaluation of sleep quality, it was used PSQI item number 9. The participants were considered “poor sleepers” when they reported “quite bad” or “very bad” sleep quality. Survey participants were asked to answer also to PSQI item number 4, estimating how many hours of sleep they get at night, both during the 4 weeks before the lockdown and the last 4 weeks before the questionnaire administration. Sleep under 6 h per night was considered insufficient. We considered a worsening in sleep quality and quantity if participants reported decreased sleep quality scores and the number of hours slept at night, respectively.

The presence of depressive symptoms was established using the 2-item Patient Health Questionnaire (PHQ-2), based on the 9-item validated scale (PHQ-9) [22]. Survey participants were asked to estimate how much they were unable to feel pleasure, and they felt down in the dumps, depressed or hopeless 2 weeks before and during the lockdown. A score of PHQ-2 ≥ 3 indicated the presence of depressive symptoms.

Anxiety symptoms were assessed using the 2-item Generalised Anxiety Disorder (GAD-2), a short version of the 7-item scale (GAD-7) [23]. The GAD-2 asked participants to assess the frequency of feeling nervous, anxious or on edge 2 weeks before and during the lockdown. The second question investigated subjects’ worrying self-control before and during the lockdown. A score of GAD-2 ≥ 3 indicated the presence of anxiety symptoms. Higher PHQ-2 and GAD-2 scores during the lockdown than before stated worsening depressive and anxiety symptoms, respectively.

Quality of life was measured using a Visual Analogue Scale (VAS) ranging between 1 (low quality of life) and 10 (high quality of life) [24]. A score of VAS < 6 indicated a low quality of life. Responders were asked to fill out the scale with reference to both before and during the lockdown. A VAS score during the lockdown lower than that reported before it defined a worsening in quality of life.

### Statistical analysis

A statistical weight has been used to ensure the representativeness of the Italian sample. Such weight was computed as a combination of two distributions of the Italian population aged 18–74: (i) sex by age (18–24, 25–34, 35–44, 45–54, 55–64, 65–74 years) by geographic area (Northwest, Northeast, Centre, South of Italy, Islands); (ii) region (the largest official area into which Italy is divided) by municipality size (5 categories). Using multiple logistic regression models, we estimated odds ratios (ORs) and corresponding 95% confidence intervals (CIs) for participants who worsened their mental health outcomes differentiating between subjects reporting reduced compared to unchanged or increased help in housework and babysitting from retired parents. We included sex, age group (< 40, 40–49, ≥ 50 years), level of education (low, intermediate, high), geographic area of residence (North, Center, South and Islands) and marital status (married, divorced/separated, widowed, single) as covariates of our model. We conducted analyses stratified by: (i) working conditions (teleworking, employed at workplace, job loss during lockdown, unemployed); and (ii) living conditions (outdoor space availability and persons per room at home). All statistical analyses were performed using SAS 9.4 (SAS Institute, Inc., Cary, NC, USA).

## Results

Table 1 reports the characteristics of the study population, including sociodemographic, exposures and outcomes distribution. Of 3156 subjects included in the analysis, 1202 (38.1%) had at least one child aged 0–14 years.

**Table 1:** Distribution of 3156 Italian adults with at least a retired parent according to selected baseline characteristics, sleep characteristics, mental health indicators (depressive and anxiety symptoms), quality of life, working and housing conditions before and during the COVID-19 lockdown. Italy, 2020

	Total	
	*N*^a^	%
*Sex*	1561	49.5
Men		
Women	1595	50.5
*Age (years)*	1058	33.5
< 40		
40–49	1062	33.7
≥ 50	1036	32.8
*Geographical area*	869	27.5
Northwest		
Northeast	639	20.2
Center	651	20.6
South	677	21.5
Islands	320	10.2
*Marital status*	2164	68.6
Married		
Divorced/separated	198	6.3
Widowed	30	0.9
Single	764	24.2
*Education*		
Low level^b^	399	12.6
Intermediate level^c^	1562	49.5
High level^d^	1195	37.8
*Help in housework*		
Unchanged	1498	47.5
Reduced	1484	47.0
Increased	174	5.5
*Help in babysitting*^e^		
Unchanged	390	32.4
Reduced	769	64.0
Increased	43	3.6
*Sleep quality*		
Poor sleep quality pre-lockdown	531	16.8
Poor sleep quality during lockdown	1226	38.9
Pre- and during lockdown % change		131.5
*Sleep quantity*		
Insufficient sleep (≤ 6 h/night) pre-lockdown	1061	33.6
Insufficient sleep (≤ 6 h/night) during lockdown	1280	40.6
Pre- and during lockdown % change		20.8
*Depressive symptoms*		
With depressive symptoms (PHQ-2 ≥ 3) pre-lockdown	422	13.4
With depressive symptoms (PHQ-2 ≥ 3) during lockdown	986	31.2
Pre- and during lockdown % change		132.8
*Anxiety symptoms*		
With anxiety symptoms (GAD-2 ≥ 3) pre-lockdown	582	18.4
With anxiety symptoms (GAD-2 ≥ 3) during lockdown	1267	40.1
Pre- and during lockdown % change		117.9
*Quality of life (QoL)*		
Low QoL (VAS ≤ 5) pre-lockdown	363	11.5
Low QoL (VAS ≤ 5) during lockdown	1302	41.2
Pre- and during lockdown % change		258.3
*Working conditions*		
Teleworking	1076	34.1
Employed at workplace	630	20.0
Job loss during lockdown	656	20.8
Unemployed	794	25.1
*Availability of outdoor space*		
Outdoor space	2423	76.8
No outdoor space	733	23.2
*People per room*		
N people per room < 1	1722	54.6
N people per room = 1	830	26.3
N people per room > 1	604	19.1

^a^Weighted

^b^No qualification, primary and secondary school certificate

^c^High school diploma

^d^University degree

^e^Based on 1202 subjects with at least one child aged 0–14 years

Forty-seven per cent of study subjects reported a reduction in help for housework from retired parents during national lockdown; for 47.5% of subjects, it did not change, while 5.5% reported an increase of help. With reference to help for babysitting, 64.0% of subjects reported a reduction; for 32.4% of subjects, it did not change, while 3.6% reported an increase.

Overall, 36.2% and 31.9% of subjects reported worsened sleep quality and quantity during lockdown, respectively. Depressive and anxiety symptoms increased in, respectively, 46.5% and 42.3%, while the quality of life was reported to have worsened during lockdown in 65.1% of the study population (Table 2).

**Table 2:** Distribution of Italians with at least a retired parent having worsened their sleep quality, sleep quantity, depressive symptoms, anxiety symptoms and quality of life during the COVID-19 lockdown, according to the changes in housework and babysitting help from parents (reduced vs unreduced*). Corresponding odds ratios** (ORs) and 95% confidence intervals (CIs). Italy, 2020

Characteristics during the lockdown	*N*	Participants worsening sleep quality (decreased self-reported sleep quality)		Participants worsening sleep quantity (decreased number of slept hours/night)		Participants worsening depressive symptoms (increased PHQ-2)		Participants worsening anxiety symptoms (increased GAD-2)		Participants worsening quality of life (decreased VAS)	
		%	OR (95% CI)	%	OR (95% CI)	%	OR (95% CI)	%	OR (95% CI)	%	OR (95% CI)
Total	3156	36.2		31.9		46.5		42.3		65.1	
*Housework help*											
Unreduced	1671	30.7	1^a^	27.5	1^a^	43.3	1^a^	37.7	1^a^	60.8	1^a^
Reduced	1485	42.3	**1.74 (1.49–2.03)**	36.8	**1.50 (1.28–1.76)**	50.2	**1.32 (1.14–1.53)**	47.6	**1.53 (1.32–1.78)**	69.9	**1.46 (1.25–1.70)**
*Babysitting help*^b^											
Unreduced	433	25.3	1^a^	24.7	1^a^	37.1	1^a^	34.3	1^a^	58.7	1^a^
Reduced	769	43.7	**2.32 (1.76–3.05)**	36.9	**1.80 (1.36–2.37)**	50.0	**1.79 (1.39–2.31)**	49.1	**1.90 (1.48–2.46)**	68.3	**1.50 (1.16–1.93)**

*The subgroups of those reporting unchanged and increased help from parents were grouped because of the smallness of the “increased help” subsample (174 and 43 subjects increasing housework and babysitting help, respectively)

**ORs and 95% CIs were estimated using unconditional multiple logistic regression models after adjustment for sex, age group (< 40, 40–49, ≥ 50), level of education (low, intermediate, high), geographic area (North, Center, South and Islands) and marital status (married, divorced/separated, widowed, single). Estimates in bold are statistically significant at 0.05 level

^a^Reference category

^b^ORs calculated on the subsample of those with at least a son aged between 0 and 14 years (*n* = 1202 subjects)

Table 2 also shows the prevalences and the adjusted ORs of worsening mental health outcomes (sleep quality and quantity, depressive and anxiety symptoms and quality of life), according to a reduced or unreduced help in housework and babysitting help from parents. Subjects reporting decreased help in housework were more likely to have worsened sleep quality and quantity (OR = 1.74, 95% CI 1.49–2.03 and OR = 1.50, 95% CI 1.28–1.76, respectively), depressive and anxiety symptoms (OR = 1.32, 95% CI 1.14–1.53 and OR = 1.53, 95% CI 1.32–1.78, respectively), and worsened quality of life (OR = 1.46, 95% CI 1.25–1.70), as compared to unreduced help. With reference to help in babysitting, decreased help was related to a worsening in sleep quality and quantity (OR = 2.32, 95% CI 1.76–3.05 and OR = 1.80, 95% CI 1.36–2.37, respectively), depressive and anxiety symptoms (OR = 1.79, 95% CI 1.39–2.31 and OR = 1.90, 95% CI 1.48–2.46, respectively), and worsened quality of life (OR = 1.50, 95% CI 1.16–1.93), as compared to unreduced help. As reported in Supplementary Table S1, excluding subjects who reported an increase in housework and babysitting help, estimates for those who experienced reduced help did not change substantially.

Table 3 reports the prevalences, the adjusted ORs and the corresponding 95% CIs of individuals worsening mental health outcomes of interest according to unreduced and reduced housework and babysitting help from parents during lockdown, by working conditions. Within workers, a worsening in mental health in subjects reporting reduced housework help from parents might be more frequent in those employed at the workplace during lockdown, as compared to those teleworking (apart from quality of life worsening); the difference was highest for worsened sleep quality, although not statistically significant (at workplace: OR = 2.18, 95% CI 1.53–3.12 vs teleworking: OR = 1.76, 95% CI 1.34–2.32). Similar patterns emerged in subjects reporting reduced help from parents in babysitting, whose risk of worsened mental health outcomes during lockdown seemed to be higher if teleworking (apart from quality of life worsening), with the largest risk difference reported for sleep quantity (at workplace: OR = 3.41, 95% CI 1.68–6.93 vs teleworking: OR = 1.83, 95% CI 1.15–2.91).

**Table 3:** Distribution of Italians with at least a retired parent having worsened their sleep quality, sleep quantity, depressive symptoms, anxiety symptoms and quality of life during the COVID-19 lockdown, according to the changes in housework and babysitting help from parents by working conditions during lockdown (reduced vs unreduced*). Corresponding odds ratios** (ORs) and 95% confidence intervals (CIs). Italy, 2020.

	Characteristics during the lockdown	*N*	Participants worsening sleep quality (decreased self-reported sleep quality)		Participants worsening sleep quantity (decreased number of slept hours/night)		Participants worsening depressive symptoms (increased PHQ-2)		Participants worsening anxiety symptoms (increased GAD-2)		Participants worsening quality of life (decreased VAS)	
			%	OR (95% CI)	%	OR (95% CI)	%	OR (95% CI)	%	OR (95% CI)	%	OR (95% CI)
	*Total*	3156	36.2		31.9		46.5		42.3		65.1	
Teleworking	*Housework help*											
	Unreduced	545	26.6	1^a^	24.6	1^a^	40.9	1^a^	35.6	1^a^	62.4	1^a^
	Reduced	530	39.0	**1.76 (1.34–2.32)**	37.7	**1.76 (1.34–2.33)**	46.5	1.22 (0.95–1.58)	45.2	**1.47 (1.14–1.91)**	70.2	**1.43 (1.09–1.86)**
	*Babysitting help*^b^											
	Unreduced	149	24.9	1^a^	25.2	1^a^	36.3	1^a^	32.9	1^a^	62.8	1^a^
	Reduced	291	41.3	**1.98 (1.25–3.15)**	38.8	**1.83 (1.15–2.91)**	48.6	1.49 (0.97–2.27)	50.1	**2.00 (1.30–3.09)**	70.7	1.37 (0.89–2.12)
Employed at workplace	*Housework help*											
	Unreduced	318	25.1	1^a^	24.3	1^a^	40.5	1^a^	33.4	1^a^	56.1	1^a^
	Reduced	312	41.5	**2.18 (1.53–3.12)**	36.1	**1.79 (1.25–2.58)**	46.7	1.31 (0.94–1.83)	47.2	**1.71 (1.22–2.40)**	63.9	1.39 (1.00–1.93)
	*Babysitting help*^b^											
	Unreduced	93	20.6	1^a^	17.5	1^a^	31.9	1^a^	31.9	1^a^	56.4	1^a^
	Reduced	173	41.6	**2.93 (1.55–5.52)**	31.9	**3.41 (1.68–6.93)**	44.7	**2.22 (1.24–3.98)**	46.4	**2.11 (1.19–3.75)**	56.1	1.01 (0.59–1.74)
Job loss during lockdown	*Housework help*											
	Unreduced	338	36.2	1^a^	30.9	1^a^	48.1	1^a^	42.8	1.00	66.3	1^a^
	Reduced	318	48.4	**1.78 (1.26–2.52)**	36.8	1.22 (0.86–1.74)	54.9	1.32 (0.95–1.84)	55.7	**1.79 (1.28–2.50)**	70.9	1.20 (0.84–1.71)
	*Babysitting help*^b^											
	Unreduced	79	35.2	1^a^	31.3	1^a^	39.2	1^a^	38.7	1^a^	60.3	1^a^
	Reduced	179	46.9	**1.91 (1.02–3.59)**	39.9	1.75 (0.90–3.40)	54.1	**2.08 (1.12–3.87)**	52.6	**2.10 (1.15–3.87)**	70.3	1.58 (0.86–2.90)
Unemployed	*Housework help*											
	Unreduced	470	35.4	1^a^	30.6	1^a^	44.4	1^a^	39.3	1^a^	58.2	1^a^
	Reduced	324	42.7	**1.44 (1.05–1.98)**	36.2	1.36 (0.98–1.89)	55.0	**1.57 (1.15–2.14)**	43.9	**1.41 (1.03–1.93)**	74.3	**1.89 (1.35–2.63)**
	*Babysitting help*^b^											
	Unreduced	112	22.9	1^a^	25.2	1^a^	40.9	1^a^	35.0	1^a^	54.0	1^a^
	Reduced	125	47.4	**3.25 (1.73–6.09)**	34.7	1.47 (0.77–2.82)	55.0	**2.05 (1.15–3.65)**	45.4	1.63 (0.91–2.92)	77.0	**3.26 (1.74–6.08)**

*The subgroups of those reporting unchanged and increased help from parents were grouped because of the smallness of the “increased help” subsample (174 and 43 subjects increasing housework and babysitting help, respectively)

**ORs and 95% CIs were estimated using unconditional multiple logistic regression models after adjustment for sex, age group (< 40, 40–49, ≥ 50), level of education (low, intermediate, high), geographic area (North, Center, South and Islands) and marital status (married, divorced/separated, widowed, single). Estimates in bold are statistically significant at 0.05 level

^a^Reference category

^b^ORs calculated on the subsample of those with at least a son aged between 0 and 14 years (*n* = 1202 subjects)

Table 4 reports the prevalences, the adjusted ORs and the corresponding 95% CIs of individuals worsening mental health outcomes of interest according to unreduced and reduced housework and babysitting help from parents during lockdown, by housing conditions. Worsening mental health in subjects reporting reduced housework help from parents during lockdown might be more frequent in those living in houses with no outdoor space, as compared to those living in houses with garden or balcony (apart from depressive symptoms worsening); the difference was highest for worsened sleep quality, even if not significantly (no outdoor space: OR = 2.15, 95% CI 1.57–2.96 vs outdoor space: OR = 1.61, 95% CI 1.34–1.93). Similar patterns emerged in subjects reporting reduced help from parents in babysitting, whose risk of worsened mental health outcomes during lockdown was higher if living in houses with no outdoor space (apart from anxiety symptoms worsening), with the largest risk difference reported for depressive symptoms (no outdoor space: OR = 2.18, 95% CI 1.20–3.98 vs outdoor space: OR = 1.78, 95% CI 1.33–2.37). About the number of persons per room, worsened mental health outcomes might be more reported in those who experienced reduced housework and babysitting help from parents during the lockdown and living with high house density (number of people per room > 1), as compared to the total sample (except the quality of life). The highest, not significant difference was assessed for sleep quantity in those reporting reduced housework help (N people per room > 1: OR = 1.97, 95% CI 1.35–2.88 vs total sample: OR = 1.50, 95% CI 1.28–1.76) and for sleep quality in those reporting reduced babysitting help (N people per room > 1: OR = 3.38, 95% CI 1.97–5.81 vs total sample: OR = 2.32, 95% CI 1.76–3.05).

**Table 4:** Distribution of Italians with at least a retired parent having worsened their sleep quality, sleep quantity, depressive symptoms, anxiety symptoms and quality of life during the COVID-19 lockdown, according to the changes in housework and babysitting help from parents during the lockdown, by housing conditions (reduced vs unreduced*). Corresponding odds ratios** (ORs) and 95% confidence intervals (CIs). Italy, 2020.

		Characteristics during the lockdown	*N*	Participants worsening sleep quality (decreased self-reported sleep quality)		Participants worsening sleep quantity (decreased number of slept hours/night)		Participants worsening depressive symptoms (increased PHQ-2)		Participants worsening anxiety symptoms (increased GAD-2)		Participants worsening quality of life (decreased VAS)	
				%	OR (95% CI)	%	OR (95% CI)	%	OR (95% CI)	%	OR (95% CI)	%	OR (95% CI)
		*Total*	3156	36.2		31.9		46.5		42.3		65.1	
		*Housework help*											
*Availability of outdoor space*	Outdoor space	Unreduced	1281	29.6	1^a^	26.7	1^a^	42.5	1^a^	37.5	1^a^	60.1	1^a^
		Reduced	1141	39.5	**1.61 (1.34–1.93)**	35.3	**1.49 (1.24–1.79)**	48.9	**1.35 (1.14–1.60)**	47.0	**1.53 (1.29–1.82)**	68.5	**1.45 (1.22–1.73)**
		*Babysitting help*^b^											
		Unreduced	345	23.5	1^a^	22.5	1^a^	35.4	1^a^	33.4	1^a^	57.5	1^a^
		Reduced	621	41.8	**2.29 (1.68–3.13)**	34.8	**1.80 (1.31–2.48)**	47.2	**1.78 (1.33–2.37)**	49.7	**2.04 (1.53–2.71)**	66.9	**1.49 (1.12–1.98)**
	No outdoor space	*Housework help*											
		Unreduced	390	34.3	1^a^	30.2	1^a^	45.8	1^a^	38.2	1^a^	63.0	1^a^
		Reduced	343	51.8	**2.15 (1.57–2.96)**	42.0	**1.56 (1.13–2.15)**	54.6	1.29 (0.95–1.76)	49.5	**1.55 (1.14–2.11)**	74.5	**1.52 (1.09–2.14)**
		*Babysitting help*^b^											
		Unreduced	88	32.4	1^a^	33.3	1^a^	43.7	1^a^	37.7	1^a^	63.2	1^a^
		Reduced	148	51.5	**2.43 (1.31–4.49)**	45.6	**2.02 (1.09–3.75)**	61.7	**2.18 (1.20–3.98)**	46.6	1.55 (0.86–2.81)	74.2	1.57 (0.84–2.91)
		*Housework help*											
*People per room*	N people per room < 1	Unreduced	951	30.2	1^a^	26.6	1^a^	43.3	1^a^	38.6	1^a^	62.1	1^a^
		Reduced	771	38.3	**1.57 (1.27–1.95)**	32.6	**1.31 (1.05–1.63)**	48.7	1.19 (0.97–1.46)	43.9	**1.33 (1.08–1.63)**	71.6	**1.60 (1.29–1.98)**
		*Babysitting help*^b^											
		Unreduced	154	20.5	1^a^	17.2	1^a^	35.6	1^a^	29.7	1^a^	59.2	1^a^
		Reduced	254	40.0	**2.65 (1.64–4.28)**	33.7	**2.61 (1.55–4.40)**	49.8	**1.90 (1.24–2.91)**	45.7	**2.03 (1.31–3.15)**	73.6	**1.88 (1.21–2.92)**
	N people per room = 1	*Housework help*											
		Unreduced	416	32.2	1^a^	29.0	1^a^	39.8	1^a^	35.6	1^a^	59.3	1^a^
		Reduced	414	47.1	**1.90 (1.40–2.57)**	40.1	**1.64 (1.20–2.23)**	49.7	**1.55 (1.16–2.07)**	49.9	**1.88 (1.40–2.52)**	68.2	**1.38 (1.02–1.86)**
		*Babysitting help*^b^											
		Unreduced	145	30.8	1^a^	29.8	1^a^	36.6	1^a^	33.3	1^a^	55.5	1^a^
		Reduced	251	45.4	**1.72 (1.08–2.75)**	34.5	1.20 (0.74–1.96)	46.4	1.45 (0.93–2.26)	50.0	**2.18 (1.37–3.44)**	68.3	**1.58 (1.01–2.48)**
	N people per room > 1	*Housework help*											
		Unreduced	304	30.1	1^a^	28.4	1^a^	44.6	1^a^	37.4	1^a^	58.9	1^a^
		Reduced	300	46.1	**2.13 (1.47–3.08)**	43.2	**1.97 (1.35–2.88)**	54.7	**1.60 (1.12–2.28)**	53.8	**1.83 (1.28–2.61)**	68.1	1.32 (0.91–1.91)
		*Babysitting help*^b^											
		Unreduced	134	25.0	1^a^	27.6	1^a^	39.2	1^a^	40.6	1^a^	61.6	1^a^
		Reduced	263	45.7	**3.38 (1.97–5.81)**	42.2	**2.08 (1.23–3.52)**	53.7	**2.42 (1.48–3.96)**	51.5	**1.91 (1.18–3.09)**	63.3	1.13 (0.70–1.82)

*The subgroups of those who affirmed unchanged and increased help from parents were grouped because of the smallness of the “increased help” subsample (174 and 43 subjects increasing housework and babysitting help, respectively)

**ORs and 95% CIs were estimated using unconditional multiple logistic regression models after adjustment for sex, age group (< 40, 40–49, ≥ 50), level of education (low, intermediate, high), geographic area (North, Center, South and Islands) and marital status (married, divorced/separated, widowed, single). Estimates in bold are statistically significant at 0.05 level

^a^Reference category

^b^ORs calculated on the subsample of those with at least a son aged between 0 and 14 years (*n* = 1202 subjects)

## Discussion

Findings from our large representative sample of Italian adults indicate the reduction in housework and childcare help from retired parents during nationwide confinement to be associated with a greater probability of worsened mental health outcomes. A trend towards stronger associations was reported in subjects employed at their workplace (as opposed to teleworking from home) and in those with poorer housing conditions.

Stay-at-home orders have been proved to effectively contain the SARS-CoV-2 spread at the population level [25, 26]. However, as confirmed by studies conducted both in Italy [20, 27–33] and in other countries [34–40], confined individuals are significantly more likely to report mental distress, anxiety and depressive symptoms, and sleep disturbances.

Within the LOST in Italy project, we have already reported [20] that during lockdown in Italy, the national-level prevalence of depressive and anxiety symptoms doubled, getting to affect more than one-third of the general adult population (respectively, 33% and 42%). Insufficient and unsatisfactory sleep and unsatisfactory quality of life registered analogous increases, reaching a mean 40% prevalence during lockdown and quite remarkable percentage changes compared to before the lockdown. We observed almost the same prevalence in our subsample, comprising adults with at least one retired parent. Overall, the available literature supports the pandemic’s independent and unprecedented effect on general population mental health, but still, scant data are available on mediators of this association.

In particular, no data have been published so far about the potential role of familial relationships and support during COVID-19 national lockdown. Our analysis explored the possible consequences of lack of familial support on households’ stability: worsened mental health in adults lacking older relatives’ help during lockdown confirms that Italian welfare deeply relies on family support [8] and raises awareness of the vulnerability of Italian families because of their dependence on older relatives [41]. Indeed, social networks and family members’ support represent crucial elements for individual wellbeing and strong predictors for mental health issues [42–46].

We observed a not significant but consistent stronger association between reduced help in childcare and worsened mental health outcomes, as compared to reduced housework help. This difference might be explained by the large proportion of Italian grandparents (about 40% according to SHARE data) [47, 48] providing essential daily childcare to their families before the pandemic, with beneficial impacts for themselves and the whole household [49]. Conversely, housework help does not include the unique emotional support intrinsic to childcare supplied by grandparents.

Among the different mental health outcomes considered, larger effects were reported for anxiety symptoms and sleep disorders, especially among adults younger than 60 years [50], as compared to depressive symptoms and worsened quality of life. Taking into consideration the survey’s timing, we can assume that mental health was mainly affected by short/mid-term effects of confinement, emergency awareness, infection fear and uncertainty about the future. In fact, as emerges from available literature, anxiety and sleep disturbances are earlier signs and predictors of mental distress [51–53].

Compared to those who shifted to teleworking during the lockdown, subjects employed at the workplace might have experienced worse mental health status decline. People working at the workplace without grandparents’ help at home might have suffered more both from heavy work shifts and exceptional workload to cope with the ongoing emergency (e.g., health workers or other essential jobs), as well as from drastically reduced available time for housework and childcare [54]. Furthermore, fear of infection at workplaces might have played a role, consistently with available evidence on the topic that associates working with emerging infectious diseases exposure risk to worse mental health outcomes [55, 56].

Reduced help in both housework and childcare was associated with a trend towards a greater risk of worse mental health in subjects reporting poor housing than in those with better living spaces. If, on the one hand, poor housing can be considered a proxy of lower socioeconomic status and a well-known risk factor for mental health [57–60], on the other hand, our findings suggest that not having an outdoor space (i.e., garden or balcony) and living in smaller houses increased the negative effect of having to look after children without help from grandparents during lockdown. Several hypotheses could be proposed to untangle this association. In compliance with the stay-at-home order, living in a house without a garden or a balcony might have caused worries about the lack of a place where children could play. Moreover, subjects were unable to enjoy the sun and the open air, this differentially adding to the risk posed on mental health by reduced help. The reduction of personal space and a daily chaotic routine, especially with children, could further explain the unease experienced by those living in small liveable spaces. Our findings are consistent with previous evidence: outdoor spaces and green elements are associated with a wide range of health benefits for all age groups, reducing stress and mental fatigue and mitigating emotional states, such as stress, depressive and anxiety symptoms [61–63]. Moreover, crowded living spaces usually stand for large families, which represent a risk factor for COVID-19-related worries [64].

Finally, as additional housework and childcare associated with COVID-19 fell mainly on women [65] and we demonstrated that grandparents’ help reduction resulted in worsened mental health, our findings could help to understand the higher odds for worsened mental health outcomes observed in young women during national lockdown [20, 32, 33, 66].

This study needs to be interpreted in light of several strengths and limitations.

To our knowledge, the LOST in Italy project is the first multidisciplinary study conducted on a large national representative sample exploring the effects of COVID-19 public health response in Italy on various behavioural risk factors, physical and mental health outcomes. The large and representative sample size distinguishes our work from other published surveys with less rigorous sampling methods and allows us to propose a fair generalisation to other settings with a similar family culture. In particular, ours is the first analysis exploring lockdown consequences on family support and mental health. The adopted study design acknowledged simulating a pre–post analysis in the context of a cross-sectional study, exploiting the first-wave nationwide lockdown as a quasi-natural experiment [67]. The use of validated evidence-based scales ensured a rigorous assessment of mental health symptoms.

Concerning limitations, the cross-sectional nature of our data does not allow us to infer causality between exposure and outcomes. Nevertheless, the direction of the nexuses is supported by social and biological plausibility. Longitudinal studies might therefore confirm results. Potential selection bias could be due to the online panel. However, a computer-assisted personal interviewing (CAPI) questionnaire was not possible during the COVID-19 lockdown, while a computer-assisted telephone interviewing (CATI) questionnaire presented limited coverage in such a relatively young population. Other limitations include potential information bias due to self-reported responses and a possible recall bias since participants were asked to report their habits and psychophysical indicators before the lockdown at the time of the interview. Furthermore, the PHQ-2 and GAD-2 scales used to assess depressive and anxiety symptoms only represent a first step screening. We also were not able to specifically take into account the representativeness of our subsamples for having at least one retired parent and children, adding this information to the statistical weight used. Yet, no major bias was derived since the whole sample was designed to be representative of the Italian population in terms of sex, age, geographic area, region and municipality size.

Our analysis informs about the importance of social networks and support within families provided by older relatives both as a resilience factor and a potential vulnerability that affects mental health outcomes [46]. This element should be considered in future public health responses, including those requiring large-scale lockdowns, quarantines or physical distancing.

First, epidemiological monitoring and screening campaigns should be timely promoted to identify people at risk of poor social and familial support and prevent further mental health problems. Secondly, informative interventions on how to deal with the mental health consequences of the pandemic and about where to get the support needed should be implemented. Finally, tailored, innovative psychosocial interventions are urged to support at-risk populations and potentially help to buffer other risk factors for mental health. Moreover, targeted community-level welfare initiatives and social policies, especially nursery schools, kindergartens, or family crèche, could limit young generations’ household dependence on older relatives’ support for childcare, with long-term beneficial consequences for all the family members. Employers could play a relevant role in financing these childcare benefits with positive consequences on workers’ mental health, especially women.

These recommendations should become part of the public health and social security strategy in general and in times of emergencies, from which mental health and related inequalities could greatly benefit [68]. Health and social services response should be designed to address mental health needs and mitigate significant long-term health costs caused by the pandemic's unprecedented stressfulness and unknown duration [69].

## Conclusion

To the best of our knowledge, this is the first sizeable original study providing representative estimates of the impact of nationwide lockdown measures on reduced familial welfare and households’ support from retired parents and, consequently, worsened mental health. We observed that national lockdown measures came along with reduced housework help supply for a large proportion of Italian adult parents who presented an increase in depressive and anxiety symptoms, sleep disorders with an unsatisfactory quality of life. Moreover, our study suggests how selected determinants and mediators, including working and housing conditions, might have worsened these changes in different behavioural, environmental and socioeconomic dimensions.

Familial support is a key determinant of mental health, the quality of which also depends on working status and conditions, house environment, and social connections. As confirmed by our results, a global, multi-level socioeconomic interdisciplinary approach involving public mental health, epidemiology, and social sciences [70] is needed to better investigate through longitudinal designs the effects of familial support changes on mental health outcomes (e.g., wellbeing, mental distress, depressive and anxiety symptoms) and to inform evidence-based family and welfare policies and prevention strategies centred on population wellbeing, within and beyond pandemic times.

## Data Availability

The datasets supporting the conclusions of this study are available from the corresponding author upon request.
